# Presarcopenia in Patients with Vitiligo: A Case Control Study

**DOI:** 10.31138/mjr.32.2.143

**Published:** 2021-05-28

**Authors:** Faiq I. Gorial, Sarah Khalaf Jehad, Salwa Faris Taha, Aseen Adil Tawfeeq

**Affiliations:** 1 Rheumatology Unit, Department of Medicine, Collage of Medicine, University of Baghdad, Iraq; 2 Rheumatology Unit, Baghdad Teaching Hospital, Medical City, Baghdad, Iraq; 3 Department of Dermatology, Baghdad Teaching Hospital, Medical City, Baghdad, Iraq; 4 Kirkuk Hospital, Kirkuk, Iraq

**Keywords:** Sarcopenia, vitiligo, sarcopenia and vitiligo

## Abstract

**Background:**

Sarcopenia is a muscle disease with significant morbidity and mortality. Vitiligo is a common autoimmune inflammatory disease which results from absence, deficiency, or dysfunction of melanocytes. Links between sarcopenia and autoimmune inflammatory processes were reported. However, no previous reports on association between sarcopenia and vitiligo were identified.

**Objective:**

To assess presarcopenia in patients with vitiligo and to evaluate the effect of sociodemographic and clinical characteristics of vitiligo patients of sarcopenia if present.

**Subject and methods:**

This case control study included 63 patients with Vitiligo and 63 apparently healthy control group matched in age and gender. Sarcopenia was diagnosed by measuring the Appendicular Lean Mass Index. Cut off point required for sarcopenia is <6 for women and <7 for men. Sociodemographic and clinical characteristics were recorded. Sarcopenia was diagnosed according to the 2018 revised European consensus on definition and diagnosis of sarcopenia.

**Results:**

Mean age of vitiligo patients was 38.7 ± 14.0 years (range: 20–69 years) and for controls 39.9 ± 11.6 years (range: 20–70 years) (p=0.604). Female were 34 (54.0%) and 29 (46.0%) males, while in the controls 30 (47.6%) were females and 33 (52.4%) males (p=0.604). Presarcopenia was significantly higher in Vitiligo compared to controls. Vitiligo increases the risk of having presarcopenia by about five-fold (OR [95%CI]=4.706[1.26–17.61], p=0.013).Only BMI was significantly negatively correlated with presarcopenia. BMI decreases the risk of having presarcopenia by odds ratio of 0.837 (0.032). other baseline characteristics had no significant impact of presarcopenia in vitiligo (P model<0.01, R^2^ =0.46 Accuracy= 0.57 AUC=0.92).

**Conclusions:**

Vitiligo was significantly positively correlated with presarcopenia and increased the risk of presarcopenia by about five-fold.

## INTRODUCTION

Sarcopenia is a progressive and generalised skeletal muscle disease associated with increased adverse outcomes including falls, fractures, physical disability, and mortality.^1^ Muscle mass and strength vary across lifetime, generally increasing with growth in youth and young adulthood, being maintained in midlife and then decreasing with ageing.^1^

Sarcopenia has long been associated with ageing, but the development of sarcopenia is now recognized to begin earlier in life. Presarcopenia corresponds to the preliminary stage of sarcopenia.^1,2^ Many factors can lead to sarcopenia^3–7^: 1) increased IL6 as proinflammatory cytokine; 2) low testosterone level and increased cortisol level, in addition to 3) low vitamin D3 level.

On the other hand, vitiligo is an autoimmune destruction of melanocytes affecting skin, hair, and mucosa.^3,4^ The main pathogenesis: 1) Increased proinflammatory cytokine (IL6)^8,9^; 2) decreased testosterone level and increased serum level of cortisol^10^; 3) very low vitamin D3 levels have been noted in patients with a variety of vitiligo vulgaris.^11,12^

Because of the shared pathogenesis of sarcopenia and vitiligo in increased serum IL6, serum cortisol level, low testosterone level, and low vitamin D3 level, so a possible link between sarcopenia and osteoporosis may be present. Accordingly, this study was designed to assess the relationship between sarcopenia and vitiligo and to evaluate the effect of sociodemographic and clinical characteristics of vitiligo patients on sarcopenia if present.

## SUBJECT AND METHODS

### Study design and settings

This case-control study was conducted at Dermatology Centre and Rheumatology Unit of Baghdad Teaching Hospital/Medical City complex from September 2018 to the end of May 2019. Out of a total of 126 Iraqi participants, 63 patients were diagnosed with vitiligo by the dermatologists and 63 apparently healthy controls matched in age and gender were enrolled in the study. Ethical approval was taken from the Department of Medicine, College of Medicine, University of Baghdad with number 30010 on 12^th^ November, 2018. Informed written consent was taken from all the participants in the study.

### Sample Selection

Patients and controls were recruited consecutively**.** Eligible patients for the study were vitiligo patients with age >18 years diagnosed by a dermatologist according to clinical features. Exclusion criteria included: Individuals who were unwilling to undergo a dual energy X-ray absorptiometry (DXA). Pregnant individuals, individuals with acute or chronic infection, diabetes mellitus, history of thyroid disease, hepatic/renal dysfunction, established malignancy, surgery,^13^ other systemic inflammatory arthritis and/or autoimmune connective tissue, and patients with history of surgery <6 months were excluded. Malnutrition was excluded by mini nutritional assessment.^14^. The controls were obtained from apparently healthy medical staff volunteers and visitors attending the outpatient clinics. These healthy controls did not have any history of autoimmune rheumatic disease.

### Clinical and Laboratory Assessment

Data were collected using a data collection sheet containing questionnaires for the patients and controls. Sociodemographic and clinical data included: age, gender, educational level and marital status, height was measured in meters (m) without shoes using a stadiometer, and weight was measured in kilograms (Kg), body mass index (BMI) was calculated according to the World Health Organization.^15^ Vitiligo evaluation included disease duration, types of Vitiligo and body surface area,^16^ and medication used. The laboratory results for complete blood count, liver function test, renal function test, erythrocyte sedimentation rate, thyroid stimulating hormone, serum level of calcium, fasting/random blood sugar, antinuclear antibody, and rheumatoid factor (if needed) were recorded.

## SARCOPENIA ASSESSMENT

### Measurement of body composition

Body composition was obtained using DXA scan, (393 rue Charies Lindbergh, 34130 Mauguio, France) machine was used for quantifying muscle mass, fat mass, lean mass, and bone mineral density (BMD) measured. The total lean mass (LM) of the extremities (total summation of muscle mass of the four limbs) was calculated and the diagnosis of sarcopenia was made via measuring the appendicular lean Mass Index (ALMI) by calculating the LM/height^2^ (kg/m2). Cut off point required for sarcopenia is <6 for women and <7 for men.^1^

### Assessment of muscle strength

Handgrip strength was measured using electronic hand dynamometer (CAMRY), tests were performed in an upright standing position, arms down by the side. Two to three test trials were performed, for both the dominant and non-dominant hand. The best trial was included in the analysis.^17^

### Physical activity measurement

Physical activity is measured by 4-m usual walking speed test with a stopwatch to measure gait timing. The cut-off speed ≤0.8 m/s is as an indicator of severe sarcopenia.^18^

### Statistical analysis

Statistical software SPSS v24 (IBM, New York, NY, USA) was used for analysis. Kolmogorov Smirnoff test was used to assess normality of continuous variables. Data was expressed as mean± SD for normally distributed continuous variables and numbers (percentages) for categorical variables. Student’s t test was used to find the difference between normally distributed continuous variables and Chi square test for categorical variables. Binary logistic regression analysis was used to assess the effect of baseline characteristics on sarcopenia. Variables included in the binary logistic regression analysis were age, gender, BMI, smoking status, vitiligo duration and type, and steroids, phototherapy, immunotherapy, and depigmentation therapy users. Missing data were checked, and basic assumptions met for logistic regression. P value < 0.05 was considered statistically significant.

## RESULTS

A total of 126 participants were included in the study, out of which 63 were vitiligo patients and 63 apparently healthy controls. The mean age of vitiligo patients was 38.7 ± 14.0 years (range: 20–69 years) which was comparable with that of the controls 39.9 ±11.6 years (range: 20–70 years) (p=0.604). Female were 34 (54.0%) and 29 (46.0%) male, while in the controls 30 (47.6%) were female and 33 (52.4%) male (p=0.476). Other baseline characteristics are shown in **Table 1**.

**Table 1.** Baseline characteristics of vitiligo patients and controls.

**Variables**	**Vitiligo=63**	**Controls=63**	**p-value**
Age (years), mean ± SD	38.7 ± 14.0	39.9 ± 11.6	0.604
Gender, n (%)			0.476
Female	34 (54.0%)	30 (47.6%)	
Male	29 (46.0%)	33 (52.4%)	
BMI (kg/m^2^), mean ± SD	27.6 ± 7.4	30.6 ± 5.7	0.012 [S]
Smoking, n (%)			0.180
Active	9 (14.3%)	6 (9.5%)	
Non-smoker	20 (31.7%)	32 (50.8%)	
Ex-smoker	8 (12.7%)	7 (11.1%)	
Passive	26 (41.3%)	18 (28.6%)	
Vitiligo duration, mean ± SD, range	13.3 ± 12.7, (0.5–64)		
Vitiligo types, n (%)			
Vulgaris	45 (71.4%)		
Universal	9 (14.3%)		
Focal	7 (11.1%)		
Acrofacial	2 (3.2%)		
BSA (%), mean ± SD	31.4 ± 32.5		
Therapy, n (%)			
Topical steroids	52 (82.5%)		
Phototherapy	22 (34.9%)		
Immune therapy (TCI)	39 (61.9%)		
Depigmentation	5 (7.9%)		

Values are means ± SD or numbers and percentages. BMI, Body mass index; kg, kilogram; m^2^, meter square; S, significant; *n,* number; P value, probability value (<0.05). TCI, Topical calcineurin inhibitors.

**Figure 1.**
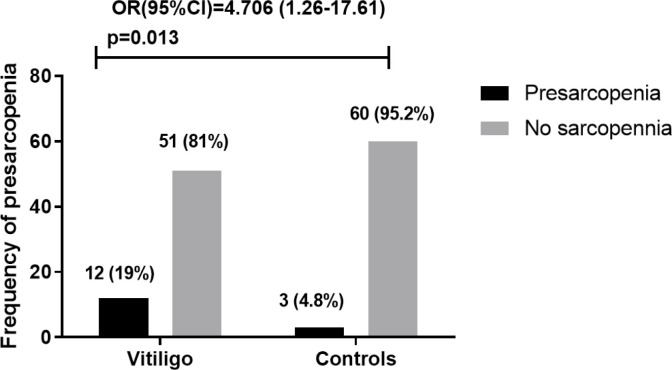
Risk of pre-sarcopenia is in vitiligo compared to controls. OR, Odds ratio; CI, confidence interval**;** P value, probability value (<0.05).

Presarcopenia was statistically significantly higher in vitiligo patients compared to controls. Vitiligo increases the risk of having presarcopenia by about five-fold (OR [95%CI]=4.706[1.26–17.61], p=0.013) as shown in **Figure 1**.

On multiple regression analysis to assess the impact of baseline characteristics on presarcopenia in vitiligo patients, we found only BMI was significantly negatively correlated with presarcopenia. BMI decreases the risk of having presarcopenia by odds ratio of 0.837 (p=0.032) as in **Table 2**.

**Table 2.** Logistic regression analysis to assess the impact of baseline characteristics of vitiligo patients on presarcopenia.

**Predictor**	**p**	**Odds ratio**	**95%CI Lower**	**Upper**
Age	0.857	0.993	0.9239	1.068
Female gender	0.113	4.867	0.6868	34.483
BMI	0.032	0.837	0.7112	0.985
Smoking	0.138	1.938	0.8080	4.650
Vitiligo duration	0.153	1.068	0.9758	1.170
Vitiligo Type	0.751	1.172	0.4401	3.121
Steroids	0.993	6.76e+8	0.0000	Inf
Phototherapy	0.298	0.283	0.0264	3.040
Immune therapy	0.685	1.487	0.2183	10.124
Depigmentation	0.997	4.22e-8	0.0000	Inf

P model<0.01 R^2^ =0.46 Accuracy= 0.57 AUC=0.92

BMI, body mass index; inf, infinity.

## DISCUSSION

The interest about sarcopenia is growing considerably. There has been a rapid growth in the scientific literature over the past years, investigating sarcopenia and related health outcomes. To the best of our knowledge, this is the first study that assessed the prevalence of sarcopenia in vitiligo patients. The current study showed that the risk of presarcopenia was increased about five-fold in vitiligo patients compared to controls. BMI was negatively correlated with presarcopenia and other baseline characteristics had no impact on presarcopenia in vitiligo patients.

Possible explanation of increased risk of presarcopenia in vitiligo may be related to several factors including autoimmunity and increased proinflammatory cytokines, psychological stress, dietary restrictions, low vitamin D level, and corticosteroids use.

Many studies showed that patients with autoimmune disease have a slightly higher risk of sarcopenia when are compared to control group; this finding may affect the quality of life and promote the increasing of morbidity in such patients.^19^ A previous Iraqi study revealed that the risk of sarcopenia was higher in rheumatoid arthritis patients in compare to healthy subjects of the same age.^20^ Another Iraqi study assessed body composition in Iraqi women with systemic lupus erythematosus revealed that there was no significant difference between body composition of lupus patients and controls.^21^ Also, it has been reported that patients with spondyloarthritis and systemic sclerosis tend to have higher risk of sarcopenia than matched controls.^22^ In systemic sclerosis, sarcopenic patients were found to have longer disease duration, worse lungs, and skin involvement.^23^ Studies indicate the intrinsic relationship between sarcopenia and diabetes mellitus pathophysiological mechanisms.^24^ Haemoglobin levels were associated with the parameters of body composition, while the decreases in muscular strength measures occur in the presence of anaemia.^25^ Glucocorticoid (GC) used in treatment of autoimmune disease could also increase muscle atrophy: GC-induced muscle atrophy is characterised by fast-twitch, glycolytic muscles, while atrophy is illustrated by decreased fibre cross-sectional area, reduced myofibrillar protein content and increased protein breakdown, and decreased protein synthesis. Increased muscle proteolysis, in particular through the activation of the ubiquitin proteasome and the lysosomal systems, is considered to play a major role in the catabolic action of GC.^26^

### Limitations

This study has some limitations: small sample size, short duration, no follow up, and inability to exclude steroids. However, this can be solved by a larger, longer, prospective study to validate the results. Despite these limitations, this study was the first study that evaluated sarcopenia among vitiligo patients.

## CONCLUSION

Presarcopenia was significantly higher in vitiligo patients compared to controls. Other sociodemographic and clinical characteristics of vitiligo patients had no significant effect on presarcopenia except for BMI, which was negatively correlated with it. These findings may suggest early screening for sarcopenia is important for diagnosis and treatment, and subsequently preventing sarcopenia complication and burden on patient quality of life.
